# Assessment of Polyphenols Bioaccessibility, Stability, and Antioxidant Activity of White Mugwort (*Artemisia lactiflora* Wall.) during Static In Vitro Gastrointestinal Digestion

**DOI:** 10.3390/foods12050949

**Published:** 2023-02-23

**Authors:** Nacha Udomwasinakun, Shikha Saha, Ana-Isabel Mulet-Cabero, Peter James Wilde, Tantawan Pirak

**Affiliations:** 1 Department of Product Development, Faculty of Agro Industry, Kasetsart University, 50 Ngamwomgwan, Lat Yao, Chatuchak, Bangkok 10900, Thailand; 2 Quadram Institute Bioscience, Norwich Research Park, Norwich NR4 7UQ, UK

**Keywords:** white mugwort, *Artemisia lactiflora* Wall., in vitro digestion, antioxidant, polyphenol profile, bioaccessibility

## Abstract

White mugwort (*Artemisia lactiflora* Wall.), a traditional Chinese medicine, has been widely consumed in different forms for health care purposes. In this study, the in vitro digestion model of INFOGEST was used to investigate the bioaccessibility, stability, and antioxidant activity of polyphenols from two different forms of white mugwort, including dried powder (P 50, 100, and 150 mg/mL) and fresh extract (FE 5, 15, and 30 mg/mL). During digestion, the bioaccessibility of TPC and antioxidant activity were influenced by the form and ingested concentration of white mugwort. The highest bioaccessibility of the total phenolic content (TPC) and relative antioxidant activity were found at the lowest P and FE concentrations, as calculated relative to the TPC and antioxidant activity of P-MetOH and FE-MetOH based on the dry weight of the sample. Post-digestion, in comparison to P, FE had higher bioaccessibility (FE = 287.7% and P = 130.7%), relative DPPH radical scavenging activity (FE = 104.2% and P = 47.3%), and relative FRAP (FE = 673.5% and P = 66.5%). Nine compounds, 3-caffeoylquinic acid, 5-caffeoylquinic acid, 3,5-di-caffeoylquinic acid, sinapolymalate, isovitexin, kaempferol, morin, rutin, and quercetin, identified in both samples were modified during digestion, yet still provided strong antioxidant activity. These findings suggest that white mugwort extract possesses a higher polyphenol bioaccessibility, showing great potential as a functional ingredient.

## 1. Introduction

White mugwort (*Artemisia lactiflora* Wall.) or Jing-Ju-Chai is an edible plant that is mainly distributed in Southeast Asia. This plant is widely used to treat menstrual and liver disorders in traditional Chinese medicine. In Thailand, the leaves and stems are used for cooking in foods and drinks or freshly consumed. White mugwort products are available on the market in several forms for different applications, such as dried leaves and dried powder for mixing with drinks and liquid extract supplements. In general, the structural components or matrix of plant-based foods can affect both the accessibility and digestibility of their bioactive compounds [1]. So far, the bioactive compounds of white mugwort have been identified by only a few studies, including diacetylenic spiroketal enol ether epoxide (AL-1) [2]; diacetylenic spiroacetal enol ethers [3]; artemisidiyne A [4]; and another twelve polyphenols, including 7-hydroxycoumarin, 7-methoxycoumarin, balanophonin, aurantiamide, aurantiamide acetate, isovitexin, kaempferol, rutin, caffeic acid ethyl ester, quercetin, methyl 3, 5-di-O-caffeoyl quinate, and methyl 3, 4-di-O-caffeoyl quinate [5]. These compounds, mainly polyphenols, exhibited promising pharmacological effects, including antioxidant, antitumor, anti-inflammatory, antiadipogenic, and antidiabetic activities [2,3,4,5,6,7,8,9]. The biological activity and health benefits of bioactive compounds, as well as polyphenols, are influenced by their chemical structures, including the number and positions of the hydroxyl groups, the presence of double bonds between carbons 2 and 3, glycosylation, and the substituents of functional groups in the rings. Studies have demonstrated that flavonoids with a higher number of hydroxyl groups exhibited stronger antioxidant activity [10,11]. Aglycone flavonoids (e.g., quercetin and apigenin) had a stronger anti-inflammatory activity compared to its glycoside form (e.g., rutin and isoquercetin) [6,12]. Flavonoids with a methoxy group at the 3-position showed the strongest antiadipogenic activity [13,14]. The key structures of bioactive compounds can be hydrolyzed and modified when exposed to the digestion process, therefore altering the bioavailability and biological activity of the compounds [15,16,17,18]. In addition, the assessment of in vitro bioaccessibility and stability are the key steps to understanding the potential health benefits of bioactive compounds. Bioaccessibility is a term describing the proportion of a compound released from a food matrix, making it available for absorption. In vitro stimulated digestion is a useful method to determine the changes and bioaccessibility of food bioactive compounds [19]. Even though polyphenols are not stable and usually degrade during the in vitro digestion process, they may still exert potential health benefits [20,21]. Apart from digestion, the food structure, as well as food processing (e.g., drying, grinding, and extracting), can alter the bioaccessibility of bioactive compounds [22,23]. The reduction of the particle size improved the release of compounds from ginseng powder during in vitro digestion [22]. Drying and grinding enhanced the bioaccessibility of flavonoids from green tea [24]. The extraction plus spray drying even enhanced the bioaccessibility of flavonoids from green tea compared to its powder and dry leaves [24]. Simulated in vitro digestions have been used to investigate the bioaccessibility, stability, and antioxidant activity of polyphenols from plant powder [20,22,25,26], fresh plants [21,27,28,29], and liquid plant extract [30,31,32,33]. There are only a few studies that have investigated the in vitro accessibility and stability of polyphenols from dried plant extracts [34,35]. More importantly, the effect of in vitro digestion on the bioaccessibility, stability, and antioxidant activity of polyphenols from white mugwort has not yet been published. Therefore, the aim of this study was to investigate the bioaccessibility and stability of polyphenols from two different forms of white mugwort: powder (P) and fresh extract (FE) at different ingested concentrations. The changes in the polyphenols and antioxidant activity during the in vitro digestion were analyzed to reveal and compare the post-ingestion potential of polyphenols from P and FE to help define the potential of using white mugwort as functional ingredients.

## 2. Materials and Methods

### 2.1. Plant Materials

#### 2.1.1. Preparation of Plant Materials

White mugwort was grown at Rairuenrom Organic Farm, Chiangrai, Thailand (19°39′25.6″ N 100°09′23.9″ E). The mature plant (40–45 days) was harvested in October 2019. The species of this plant was confirmed by an expert and registered as Artemisia lactiflora Wall. (BK No.070334) in The Forest Herbarium, Department of National Parks, Wildlife and Plant Conservation, Thailand. Plant materials used in this study consisted of fresh aerial parts (leaves and stems) and dried powder from white mugwort. The fresh aerial part of white mugwort was dried in a parabola dome at 65 ± 5 °C for 48 h. The dried plant was ground and sieved through 100 mesh screens to obtain the dried powder sample (P). The proximate compositions of the fresh and dried powder of white mugwort were determined [9]. The fresh extract was prepared using the solvent extraction method. Cleaned fresh aerial parts of white mugwort were blended using a high-speed electric blender (FDP623WH, KENWOOD) for one minute. The blended sample was extracted using 95% ethanol (Sigma-Aldrich, Darmstadt, Germany) in a volumetric flask covered with aluminum foil and homogeneously mixed for 2.5 min at 25 ± 1 °C using a magnetic stirrer. The mixture was filtered using 0.45 µm filter paper. A rotary evaporator was used to remove the solvent. Finally, the solvent-free extract was dried using a freeze drier to obtain dried fresh extracts (FE) and kept at −80 °C until the analysis.

#### 2.1.2. Quantification of Polyphenols in Original Starting Materials

The undigested P and FE samples underwent a methanol extraction to quantify the polyphenol content to be used as reference values for the release of polyphenols from the digested samples. The starting material (P and FE) was diluted in 70% methanol (Sigma-Aldrich, Darmstadt, Germany) at a concentration of 100 mg/mL (to maximize the measurement accuracy) and placed in an Eppendorf. After centrifugation (Eppendorf MixMate^®^, 10 min, 25 °C, 5000 rpm), the aqueous phase was collected, and the insoluble fraction was further extracted using another 1 mL of 70% methanol by the same method as described above. The supernatants from each extraction were pooled together for each sample; then, the methanol extracts of P and FE, namely P-MetOH and FE-MetOH, were analyzed for their polyphenol contents (−2.2.4) and used as the reference values [28].

### 2.2. Simulated Static In Vitro Gastrointestinal Digestion

The in vitro gastrointestinal digestions were performed following the standardized INFOGEST protocol of static in vitro gastrointestinal digestion model [36] with minor modifications. The activity of all digestive enzymes used in this study was determined prior to the experiment to meet the recommended activity. All simulated digestive fluids, simulated saliva fluids (SSF), simulated gastric fluids (SGF), and simulated intestinal fluids (SIF) were prepared according to the INFOGEST protocol [36]. The digestion was done in separate 50 mL plastic centrifuge tubes for the gastric phase (G) and gastrointestinal phase (I). To study the effect of white mugwort concentration on the total phenolic content (TPC) released during in vitro digestion, samples were prepared prior to the digestion by mixing with Milli-Q water at different concentrations (P at 50, 100, and 150 mg/mL and FE at 5, 15, and 30 mg/mL). Briefly, 2.5 g of sample was mixed with 2.5 mL of SSF and incubated for 2 min. SGF was then added to the oral mixture and adjusted to pH 3.0 using 1.0 M HCl before adding the pepsin solution (2000 U/mL) (SLCG8343, Sigma-Aldrich, Gillingham, UK). The final volume of gastric digestion was 10 mL. The mixture was incubated at 37 °C in a rotator at 170 rpm. After 120 min of gastric digestion (G120), the samples were collected and adjusted to pH 7.0 using 1.0 M NaOH. Moving to the intestinal phase, the gastric mixture was mixed with SIF and adjusted to pH 7.0 using 1.0 M NaOH (I0) before adding the pancreatin solution (trypsin activity 100 U/mL) (SLBV6830, Sigma-Aldrich, Gillingham, UK) and bile solution (0.15 mM bile salts) (SLCD0888, Sigma-Aldrich, UK). The final volume of intestinal digestion was 20 mL. The mixture was incubated at 37 °C in a rotator at 170 rpm for another 120 min of the intestinal phase (I120). To stop the enzyme activity and prevent the degradation of polyphenol in an alkaline condition, the samples were adjusted to pH 2.0 using 1.0 M HCl at the end of the intestinal phase. All samples were centrifuged at 5000 rpm for 10 min. The supernatants (soluble, bioaccessible fraction) were snap-frozen in dry ice and kept at −80 °C until the analysis of the total phenolic content (TPC) (Section 2.2.1), antioxidant activity (Section 2.2.2), and polyphenol profile (Section 2.2.3). The bioaccessibility of the TPC and relative antioxidant activity were calculated according to Equations (1) and (2) [37]:

(1) $$\mathrm{Recovery}\;\mathrm{or}\;\mathrm{Bioaccessibility}\;\mathrm{of}\;\mathrm{TPC}\;(\%)=\frac{{\mathrm{TPC}}_{\left(\mathrm{solule}\;\mathrm{fraction}\right)}}{{\mathrm{TPC}}_{\left(\text{P-MetOH}\;\mathrm{or}\;\text{FE-MetOH}\right)}}\times 100$$

(2) $$\mathrm{Relative}\;\mathrm{antioxidant}\;\mathrm{activity}\;(\%)=\frac{{\mathrm{Antioxidant}\;\mathrm{value}}_{\left(\mathrm{solule}\;\mathrm{fraction}\right)}}{{\mathrm{Antioxidant}\;\mathrm{value}}_{\left(\text{P-MetOH}\;\mathrm{or}\;\text{FE-MetOH}\right)}}\times 100$$

The experiments were done in three independent replications. The background condition represents the treatment that passed through all digestion processes without white mugwort. In addition, all data of the background conditions were subtracted from the data of all digestion treatments to avoid false detection. 

#### 2.2.1. Analysis of Total Phenolic Content (TPC)

The method of Karabegovic et al. [38] was used for the determination of the total phenolic content. The reaction was done in a 96-well plate. Briefly, 20 μL of sample was mixed with 100 μL of Folin–Ciocalteu reagent (10%, *v*/*v*) (Merck, Darmstadt, Germany) and 80 μL of Na_2_CO_3_ solution (7.5%, *w*/*v*) (Sigma-Aldrich, Darmstadt, Germany). After 30 minutes of incubation in the dark, the absorbance was measured at 760 nm using a Molecular Devices, LLC VersaMax plate reader (San Jose, CA, USA). Distilled water was used as a blank. Gallic acid (5–500 µg/mL) (Sigma-Aldrich, Gillingham, UK) was used to create the standard curve with a correlation coefficient at 0.9956. The TPC values were expressed as milligram gallic acid equivalents per milliliter (mg GAE/mL).

#### 2.2.2. Analysis of Antioxidant Capacity

The free radical scavenging activity was determined using the DPPH assay of Hwang et al. [39]. The assay was done using a 96-well plate. Briefly, 50 μL of the sample was mixed with 150 μL of DPPH ethanolic solution (0.1 mM) (Alfa Aesar Massachusetts, USA). The reaction mixture was incubated for 30 min at room temperature. The absorbance of the resulting solution was read at 517 nm using a VersaMax plate reader. Distilled water was used as a blank. Gallic acid (range of 2.5–100 ppm) was used to create the standard curve with a correlation coefficient at 0.9367. The DPPH radical scavenging activity was expressed as milligram gallic acid equivalents per milliliter (mg GAE/mL).

The ferric-tripyridyltriazine (Fe^3+^)-reducing activity was evaluated using the FRAP assay of Ketnawa et al. [40]. The reaction was done using a 96-well plate. The FRAP reagent was freshly prepared using 0.3 M acetate buffer (pH 3.6) (Sigma-Aldrich, Gillingham, UK) plus 10 mM TPTZ in 40 nM HCl (Sigma-Aldrich, Gillingham, UK) and 20 nM FeCl_3_ (Sigma-Aldrich, Gillingham, UK) at a ratio of 10:1:1, *v*/*v*. The reagent was incubated at 37 °C for 30 min prior to the analysis. Briefly, 20 μL of sample was mixed with 120 μL of FRAP reagent. The mixture was incubated in the dark for another 30 min before recording the absorbance at 593 nm using a VersaMax plate reader. Distilled water was used as a blank. Fe^2+^ in the range of 100–1000 μmol/mL was prepared from iron (II) sulfate heptahydrate (FeSO_4_·7H_2_O) (Sigma-Aldrich, Gillingham, UK) and used to create the standard curve with a correlation coefficient at 0.9903. The FRAP values were expressed as the mole of Fe^2+^ equivalents per milliliter (mol Fe(II)/mL).

#### 2.2.3. Identification of Polyphenol Profile by HPLC-DAD/MS

The polyphenol composition was determined according to the method of Zhang [33], with minor modifications. A HPLC analysis was conducted on an Agilent 1100 series system instrument (Agilent Technologies 1100 Series LC) equipped with a quaternary pump (G1311A Quat Pump), an automatic sampler (G1329A ALS), and a diode array detector (G1315A DAD). Chromatographic separation was done on a Phenomenex Luna 5u C18 (2) column (250 × 4.6 mm, 5 μm). The results were acquired by OpenLab CDS software (Agilent Technologies). A HPLC-DAD analysis was performed at 30 °C, with a flow rate of 0.8 mL/min and injection volume of 20 μL. Acetic acid at 3%, *v*/*v* (solvent A) and acetonitrile (solvent B) were used as the solvent mobile phase composition. The gradient elution profile of solvent B was as follows: 0.00–5.00 min, 0–8.5%; 5.00–16.50 min, 8.5–2.0%; 16.50–35.00 min, 2.0–18%; 35.00–50.00 min, 18–20%; 50.00–65.00 min, 20–30%; 65.00–70.00 min, 30–0%. The absorbance was recorded at 280 nm. All tested samples were filtered through a 0.45 μm membrane filter before the analysis. The standard compounds used for the chromatographic peak identification were as follows: 3-caffeoylquinic acid (3-CQA), 5-caffeoylquinic acid (5-CQA), 3,5-di-caffeoylquinic acid (3,5-diCQA), isovitexin, kaempferol 3-o-β-rutinoside, morin, rutin, quercetin, and quinic acid (Sigma-Aldrich, Gillingham, UK). The correlation coefficient values achieved from the created standard curves were in the range of 0.09938 to 0.9996.

The identification was also done based on byproduct ion monitoring using 6490 Triple-Quad LC-MS/MS (6490 MS/MS, Agilent Technologies, CA, USA) equipped with a diode array detector (G4212A 1290DAD) and Phenomenex Luna C18 column (250 × 4.6 mm, 5 μm). The MS conditions of Sivapalan et al. [41] were used for the analysis. The LC conditions, flow rate, ejection volume, solvent type, and gradient profile were as described above. Each compound was identified using the mass spectrum and compared with the literature data [42,43]. The recovery and bioaccessibility of each compound were calculated according to Equation (1).

### 2.3. Statistical Analysis

All data are presented as the mean ± SD obtained from three individual experiments. The statistical analysis was done using the SPSS Statistics for Windows, Version 12.0 (SPSS Inc, SPSS Inc., Chicago, IL, USA). The different mean values were analyzed using the independent samples *t*-test and one-way analysis of variance (ANOVA). The Duncan Multiple Range Test (DMRT) was applied for mean comparisons. The significant differences were considered at *p* ≤ 0.05 (95% significance interval). Pearson’s bivariate correlation was used to determine the correlation coefficient (r).

## 3. Results and Discussion

### 3.1. Changes in TPC and Antioxidant Activity of White Mugwort during In Vitro Gastrointestinal Digestion

**Figure 1:**
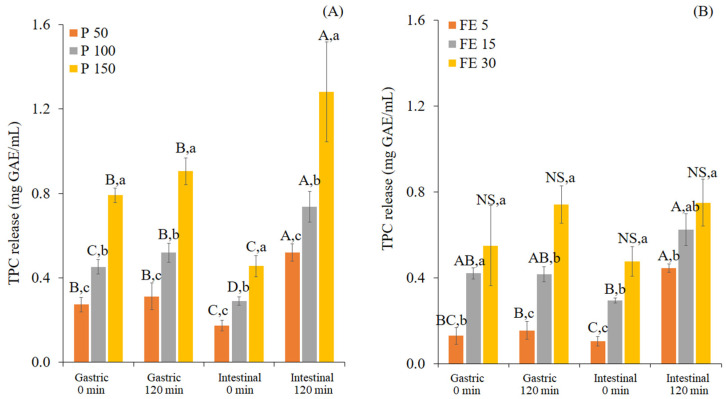
Changes in the proportionate total phenolic content released (mg GAE/mL) from white mugwort powder at different concentrations (P 50, 100, and 150 mg/mL) (**A**) and white mugwort extract at different concentrations (FE 5, 15, and 30 mg/mL) (**B**) during in vitro gastrointestinal digestion. Values with different letters (A–D) within the same treatment are significantly different (*p* ≤ 0.05). Values with different letters (a–c) within the same digestion phase are significantly different (*p* ≤ 0.05). A *p*-value higher than 0.05 is represented by NS (no statistically significant difference).

The measures of TPC released from P (50, 100, and 150 mg/mL) and FE (5, 15, and 30 mg/mL) during in vitro gastrointestinal digestion were analyzed and are shown in Figure 1.

When the samples were exposed to the in vitro digestion process, the TPC measured at G0 were found to be 0.3, 0.5, and 0.8 mg GAE/mL of P (50, 100, and 150 mg/mL) and 0.1, 0.4, and 0.6 mg GAE/mL of FE (5, 15, and 30 mg/mL). In addition, the TPC did not appear to increase during gastric digestion, since there was no significant difference (*p* > 0.05) of the TPC between G0 and G120 of both samples. However, the sudden transition from acidic conditions at G0 to alkaline conditions at I0 caused a slight decrease of the TPC (Figure 1A,B). This could be due to the degradation of polyphenols under alkaline conditions [21,25,26]. The presence of pancreatic enzymes with bile salts in the intestinal phase appeared to induce a further release of the TPC, especially from P. These findings indicate the stronger impact of enzymatic digestion on TPC release compared to the pH change [44]. At the end of digestion (I120), the TPC release was increased to the levels of 0.5, 0.7, and 1.3 mg GAE/mL of P (50, 100, and 150 mg/mL) and 0.4, 0.6, and 0.8 mg GAE/mL of FE (5, 15, and 30 mg/mL), respectively. The increase of TPC release in the intestinal phase supports the idea that the intestinal condition enhances the solubility of polyphenol compounds via the presence of pancreatic enzymes and/or bile salts. This was confirmed by Yang et al. [45], who found that bile acids can improve the TPC released from kale extract by interaction through hydrogen bonding.

**Figure 2:**
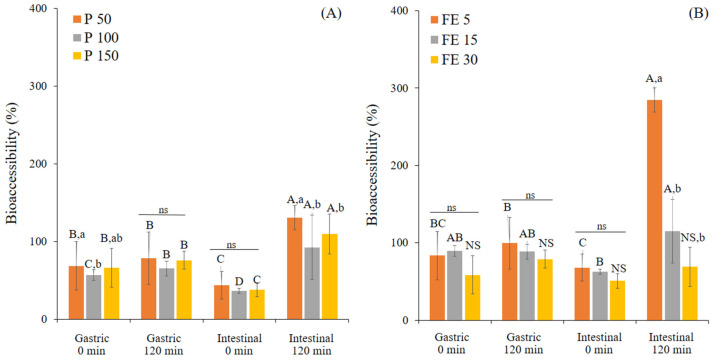
Bioaccessibility (%) of the TPC (compared to methanolic extraction) from white mugwort powder at different concentrations (P 50, 100, and 150 mg/mL) (**A**) and white mugwort extract at different concentrations (FE 5, 15, and 30 mg/mL) (**B**) during in vitro gastrointestinal digestion. Values with different letters (A–D) within the same treatment are significantly different (*p* ≤ 0.05). Values with different letters (a,b) within the same digestion phase are significantly different (*p* ≤ 0.05). A *p*-value higher than 0.05 is represented by NS or ns (no statistically significant difference). Values were calculated based on the TPC per gram of dry weight of the sample.

The current study first estimated the bioaccessibility of TPC from white mugwort (Figure 2). The data regarding the bioaccessibility of the TPC were calculated relative to the starting TPC value of P-MetOH and FE-MetOH, assuming the methanol was the best solvent that could extract a 100% of the measurable TPC in the starting sample. It is important to note that the biaccessibility values were calculated based on the TPC per gram dry weight of the sample (Figure S1). Initially, the TPC of FE-MetOH (125.4 ± 12.6 mg GAE/g DW) was 3.9 times higher than P-MetOH (31.81 ± 0.73 mg GAE/g DW) (Figure S1). Therefore, the higher TPC is normally found in plant extract (FE) compared to its original form (P) based on the dry weight of a one gram sample.

At G120, the bioaccessible TPC of P and FE were in the range of 65.4% to 78.5% and 78.8% to 99.5%, respectively, showing no significant difference (*p* > 0.05) to those found in G0. Similar to the TPC release, the bioaccessibility of TPC was significantly increased after I120. The different ingested concentrations of P and FE had a significant effect on the bioaccessibility. After I120, the bioaccessibility of P at 50, 100, and 150 mg/mL were 130.7%, 92.7%, and 109.8%, respectively, while FE at 5, 15, and 30 mg/mL had 287.7%, 115.1%, and 69% bioaccessibility, respectively. It is worth emphasizing that P and FE at the lowest ingested concentrations had the highest TPC released. This might be explained by the mass transfer principles, since the solid-to-solvent ratio is one of the factors affecting the extraction of polyphenols from the solid matrix during digestion, where the extraction efficiency is increased with increasing of the proportion of the solvent [21,46]. The methanol extractions of P-MetOH and FE-MetOH were measured at the concentration of 100 mg/mL, which was much higher than the ingested concentrations of P and FE. Therefore, this may accentuate this concentration effect and explain the apparently high bioaccessibility values. Nevertheless, the relative values for bioaccessibility during digestion and between samples still indicate the ability of the different digestion phases to release polyphenols from the samples. Furthermore, bioaccessibility above 100% indicates a stronger ability of a particular gastrointestinal digestion phase to chemically and physically enhance the solubility of TPC from FE and extracting TPC from the cell wall structure of P. Other studies also reported the increasing release rate of bioactive compounds from the solid matrix of powder samples over time during in vitro digestion [20,22,26]. The greater bioaccessibility percentage in FE over P implies the ability of the solvent extraction to enhance the bioaccessibility of the TPC. These findings are consistent with the study of Oh [24], who reported the 11-times higher bioaccessibility of polyphenols in green tea extract than powdered green tea. It is noteworthy that the stronger effect of the ingested concentration on bioaccessible TPC was found in the extract compared to the powder. Purified extracts, on the other hand, contain high quantities of the key compounds extracted from the intact cell wall; therefore, it ought to impart higher bioaccessiblity values than the powdered form, where the bioactive compounds are still interacting with the cell wall present in the sample. On the other hand, the gastrointestinal digestion also causes the negative impact on the stability of polyphenols, such as pH and electrolyte changes induce the structural modification and degradation of polyphenols [21,25,26]. Furthermore, binding with bile salts and interactions with digestive enzymes can induce the formation of insoluble complexes [20,47], which could decrease the bioaccessibility, as found in some treatments in this study.

**Figure 3:**
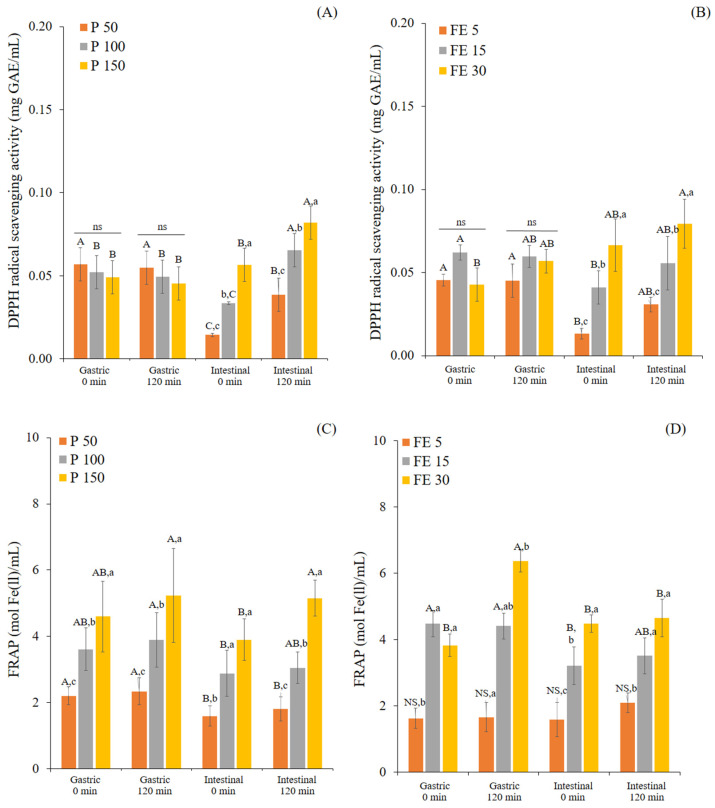
Changes in the DPPH scavenging activity (mg GAE/mL) (**A**,**B**) and FRAP value (mol Fe(ll)/mL) (**C**,**D**) of white mugwort powder (P 50, 100, and 150 mg/mL) and white mugwort extract (FE 5, 15, and 30 mg/mL) during in vitro gastrointestinal digestion. Values with different letters (A–C) within the same treatment are significantly different (*p* ≤ 0.05). Values with different letters (a–c) within the same digestion phase are significantly different (*p* ≤ 0.05). A *p*-value higher than 0.05 is represented by NS or ns (no statistically significant difference).

Polyphenols are powerful antioxidative components that can protect biomolecules from oxidative damage, which contributes to their beneficial effects in the prevention of several diseases [48]. Therefore, antioxidant activity is the primary screening method for further biological activity. Bioactive compounds can only exert their bioactivity after being released from the food matrix [25]. Hence, the accessible fractions (supernatant) of P and FE after G120 and I120 were analyzed for their antioxidant activity using DPPH radical scavenging and the FRAP assay (Figure 3).

**Figure 4:**
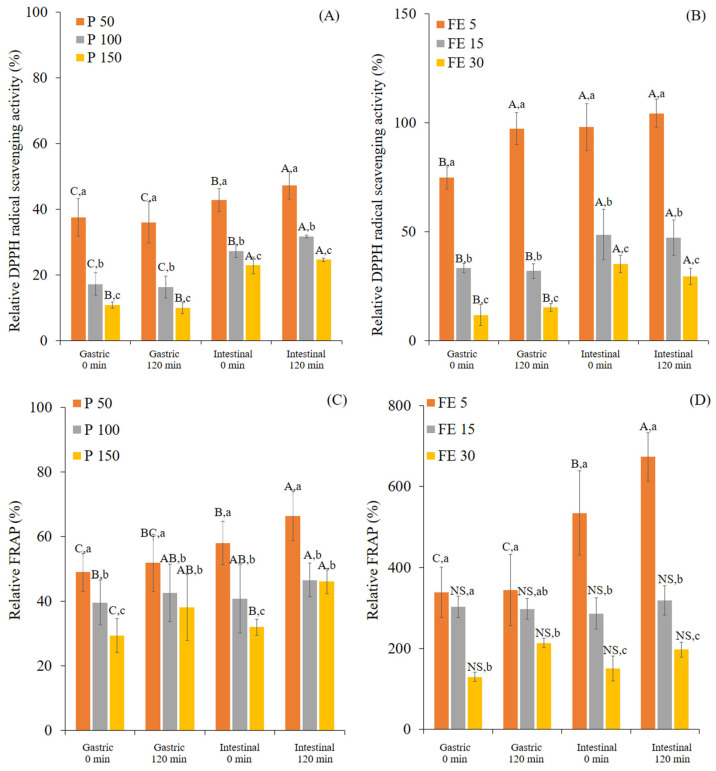
The relative DPPH scavenging activity (%) (**A**,**B**) and FRAP value (%) (**C**,**D**) of white mugwort powder (P 50, 100, and 150 mg/mL) and white mugwort extract (FE 5, 15, and 30 mg/mL) during in vitro gastrointestinal digestion. Values with different letters (A–C) within the same treatment are significantly different (*p* ≤ 0.05). Values with different letters (a–c) within the same digestion phase are significantly different (*p* ≤ 0.05). A *p*-value higher than 0.05 is represented by NS (no statistically significant difference).

During digestion, the DPPH and FRAP values were related to the TPC release, showing dose-dependent behavior. Except for the DPPH values in the gastric phase of both samples. The various ingested concentrations of P and FE had no significant differences in DPPH values at times 0 and 120 min in the gastric phase (*p* > 0.05). The sudden decrease of the DPPH and FRAP values at I0 were similar to the decrease of the TPC values caused by the degradation of polyphenols owing to the pH change from acidic conditions (pH 3) to alkaline conditions (pH 7), as well as the electrolyte changes [20,21,25]. In order to indicate the effect of the ingested concentration on the antioxidant activity, the antioxidant activity was calculated relative to the DPPH and FRAP values of P-MetOH and FE-MetOH, assuming the methanol extracted 100% of the measurable antioxidant activity (Figure 4).

Similar to the bioaccessibility values, the relative antioxidant activity was calculated based on the antioxidant activity per gram of dry weight of the sample (Figures S2 and S3). Initially, the DPPH radical scavenging activity of P-MetOH and FE-MetOH were 13.0 ± 1.0 and 52.9 ± 1.0 mg GAE/g DW, respectively, whereas the FRAP values of P-MetOH and FE-MetOH were 0.37 ± 0.0 and 0.40 ± 0.0 mol Fe(II)/g DW, respectively. After digestion, the antioxidant activity of P and FE remained. The highest relative antioxidant activity was found at the lowest ingested concentration of both samples, indicating that the ingested concentration of P and FE affected not only their TPC release but also their antioxidant activity. After I120, the relative scavenging activity of P and FE were increased significantly higher than those found in G120. However, the significant increase of relative FRAP from G120 to I120 was only found at the lowest ingested concentrations (P 50 and FE 5 mg/mL). The increase of antioxidant activity during the intestinal phase could have been due to the ability of intestinal digestive enzymes and bile salts to extract or solubilize phenolics from the solid matrix, resulting in the increase of the TPC and bioactivity [20,22,26]. The highest relative scavenging activity found in P50 and FE5 mg/ml was 47.3% and 104.2%, respectively, while the highest relative FRAP found in P50 and FE5 mg/ml was 66.5% and 673.5%, respectively. These results also indicated the stability of antioxidative components of white mugwort during in vitro digestion conditions. The very high relative DPPH and FRAP values for FE suggested the concentration effect as already described for the very high bioaccessibility of the TPC. This is probably because the FE-MetOH sample was measured at a much higher sample concentration than used in the digestion.

The original FE sample had a stronger antioxidant capacity than P both in the DPPH radical scavenging and FRAP assays (Figures S2 and S3). The antioxidant activity of both P-MetOH and FE-MetOH were correlated with their TPC. The antioxidant activity is generally correlated with the number of hydroxyl groups on the polyphenol structure, as reported previously [33,38,49]. Therefore, the overall antioxidant activity will be the result of both the concentration and molecular structure of polyphenols. In this study, the Pearson correlation coefficient (r) analysis revealed that TPC was positively correlated with the antioxidant activity assays: DPPH radical scavenging (r = 0.965) and FRAP (r = 0.638), respectively, indicating a very strong and strong correlation [50,51]. The stronger correlation of the TPC with DPPH over FRAP was also reported in previous studies [26,38,49,52]. The different correlation levels could be explained by the different reaction mechanisms of the assays. The TPC assay is a measure of the total reducing capacity of the tested sample, mainly based on the electron transfer reaction between the Folin–Ciocalteu reagent and reducing compounds [53,54]. The DPPH assay is based on hydrogen atom transfer and electron transfer, while FRAP determines the ability to reduce ferric (Fe^3+^) to ferrous (Fe^2+^) based on the electron transfer reaction [52].

### 3.2. Changes of Polyphenol Profile during In Vitro Gastrointestinal Digestion

**Figure 5:**
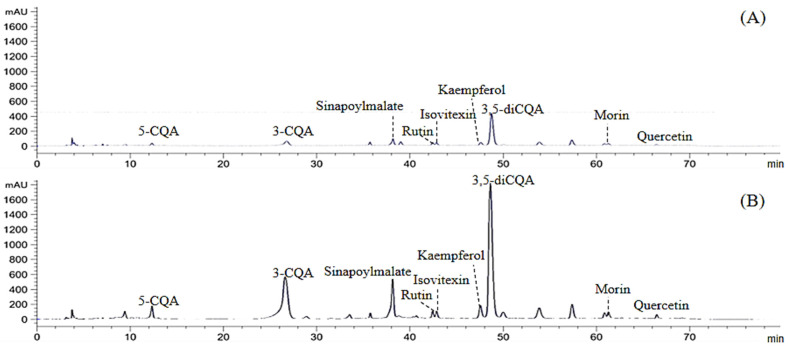
HPLC chromatograms of the polyphenol profile of (**A**) dried powder (P-MetOH) and (**B**) fresh aerial part extract (FE-MetOH) from white mugwort at 280 nm. The content of each compound is as listed in Table 1 and Table 2.

The polyphenol compositions of P and FE were determined from the methanol extraction of both samples, P-MetOH and FE-MetOH (Figure 5). The identification and quantification of the polyphenols were performed by the analysis of their retention times and compared with the standard compounds. Only sinapoylmalate at the retention time of 38.2 min with λmax at 280 nm was confirmed by LC-MS, according to the mass spectrum (ESI-MS-m/z + 341[M + H]^+^, 339[M − H]^−^, 363.07[M + Na]^+^). Due to the lack of standard compound, the concentrations of sinapoylmalate between P and FE were compared based on the relative peak area detected by LC-DAD (Table S1).

The different quantity of eight key phenolic compounds, including three phenolic acids (5-caffeoylquinic acid (5-CQA), 3-caffeoylquinic acid (3-CQA), and 3,5-di-caffeoylquinic acid (3,5-diCQA)) and five flavonoids in both aglycone (quercetin, kaempferol, and morin) and in glycosidic forms (rutin and isovitexin), were found in both samples. FE-MetOH had higher contents of all the compounds compared to P-MetOH. Chlorogenic acids or caffeoylquinic acids (CQAs) were found to be the predominant phenolic groups in white mugwort. The variations of these CQA isomers were found in FE (3-CQA > 3,5-diCQA > 5-CQA: 22.26 > 19.85 > 0.26 mg/g DW) and P (3,5-diCQA > 3-CQA > 5-CQA: 4.58 > 1.40 > 0.04 mg/g DW). From these results, 3-CQA and 3,5-diCQA were found to be the most abundant CQA isomers in white mugwort, and the total amount of three chlorogenic isomers detected in P and FE were 6.02 and 42.37 mg/g, respectively. Although CQAs are the well-known compounds found in coffee (4–11 mg/g DW) [55], a higher total chlorogenic acid content (3.91–514.65 mg/g DW) was reported in different Artemisia species (e.g., mugwort (*Artemisia vulgaris*) and wormwood (*Artemisia absinthum*)), depending of the species and different growing environments [56,57]. Moreover, 3,5-diCQA following 3-CQA and 5-CQA were reported as the most abundant chlorogenic isomers found in mugwort (*Artemisia vulgaris*), similar to the findings in this study [56]. Therefore, plants in the Artemisia genus could be alternative sources of chlorogenic acids. Five flavonoids, including kaempferol, morin, rutin, isovitexin, and quercetin, were also detected in the white mugwort samples with various quantities ranging from 0.12 to 5.34 mg/g DW. Moreover, the presence of these flavonoids has previously been reported in white mugwort and suggested to be responsible for the biological activity of white mugwort (e.g., anti-inflammatory and antiadipogenic activity) [5,9,58]. Due to the fact that the bioavailability and biological activity of individual polyphenols are based on their molecular structures, the changes in the polyphenol composition during in vitro digestion need to be investigated [1]. In this study, the changing concentrations of eight compounds were analyzed from the soluble fraction (accessible fraction) obtained during in vitro gastrointestinal digestion. The background conditions, including digestive fluids and enzymes, were found to interfere in the interpretation of the polyphenol profile of digesta. Therefore, the HPLC-DAD data of the background conditions were subtracted from the digesta before calculating the content of each compound, as shown in Table 1 and Table 2. In Table 1, gastric digestion released lower amounts of polyphenols compared with P-MetOH. The most accessible compound from P after gastric digestion was 5-CQA. Apart from the compounds identified in P-MetOH, gastric digestion released quinic acid from P. The transition from the gastric phase to intestinal phase caused the decrease and even disappearance of all compounds, except for quinic acid. The bioaccessibility of each compound was calculated relative to P-MetOH. The most accessible compound after intestinal digestion was rutin (32–39%). Similar to these findings, the high stability of rutin over kaempferol and quercetin was also found by Ed Nignpense [44], who reported the 60.8% bioaccessibility of rutin after intestinal digestion of polyphenol-rich purple rice. The bioaccessibility of all the compounds was related to the ingested concentration of P, except for quinic acid, where the highest content of quinic acid was found at the lowest ingested concentration (P50 mg/mL), consistent with the bioaccessible TPC (Figure 2). The in vitro digestion released greater amounts of phenolic compounds from FE compared to P. In Table 2, the ingested concentration of FE altered the bioaccessibility of each compound. In the gastric phase, the most accessible compound of FE 5 mg/mL was isovitexin (167%), followed by rutin (122%) and quercetin (106%), whereas kaempferol (210% and 249%) was the most accessible compound of FE 15 and 30 mg/mL. The bioaccessibility greater than 100% indicates the stronger efficiency of gastrointestinal digestion to the extract and release of the compound than the organic solvent (methanol) due to the actions of digestive enzymes and pH change during the digestion process [44]. The high bioaccessibility of the TPC may also be influenced by the high sample concentration used for FE-MetOH extraction, as discussed earlier. The transition from gastric to intestinal conditions results in the decrease of all the compounds, except for quinic acid, where the highest content of quinic acid (6.1 mg/g DW) was found at the lowest ingested concentration (FE 5 mg/mL) consistent with the bioaccessible TPC (Figure 2). These findings indicate the impact of the digestion process on the release of quinic acid, since the same phenomenal was also found in P. Apart from the ability of the digestion process to release quinic acid from P and FE, the increase of quinic acid during digestion could be due to the degradation of chlorogenic acids (3-CQA, 5-CQA, and 3,5-diCQA). Chlorogenic acids, the water-soluble esters between quinic acid and hydroxycinnamic acids, easily degrade at pH 5.0–9.0 (37 °C) and were found to be hydrolyzed and absorbed in the small intestine, which can be detected as the simpler phenolics such as caffeic, ferulic, and quinic acids [15,16,17,18]. Previous studies have indicated the cleavage of chlorogenic acids into caffeic and quinic acids via metabolic pathways [59,60]. It is noteworthy that the intestinal digestion of FE 5 mg/mL increased 5-CQA (4.3 mg/g DW) to the final bioaccessibility at 1671%. However, this did not occur at the higher concentrations. The great percentage of bioaccessible TPC after I120 of FE 5 mg/mL (Figure 2) could be due to the increase of 5-CQA and quinic acid. Even though the polyphenols of white mugwort were not stable during the digestion process and transformed to smaller molecules, they still showed strong antioxidant activity.

## 4. Conclusions

This is the first time that the bioaccessibiltiy, stability, and antioxidant activity of polyphenols from white mugwort (*Artemisia lactiflora* Wall.) have been investigated during in vitro gastrointestinal digestion. The polyphenols were released from P and FE during the digestion process. Our study suggested the impact of the ingested concentration and form of the samples on the bioaccessibility of the TPC and antioxidant activity. The bioaccessibility of the TPC and relative antioxidant activity were influenced by the forms and ingested concentrations of white mugwort. The lowest ingested concentrations of the samples were more effective at promoting high bioaccessibility and relative antioxidant activity. Moreover, FE or the extract form provided higher bioaccessibility than P. The present study identified nine compounds in the group of phenolic acids and flavonoids in both P and FE. Most of the compounds were not stable during the digestion process. The polyphenols were significantly decreased or disappeared during intestinal digestion. Quinic acids were found after the digestion process due to the degradation of chlorogenic acids (3-CQA, 5-CQA, and 3,5-diCQA), which still provided strong antioxidant activity. These findings suggest that the processing, such as grinding and extracting, could improve the bioaccessibility of polyphenols. Extraction improved the bioaccessibility, showing the post-digestion potential of FE as a functional ingredient. The further investigation of bioavailability of white mugwort polyphenols is required in future in vivo studies.

## Figures and Tables

**Table 1:** The changes of polyphenol compounds of white mugwort powder during in vitro gastrointestinal digestion.

Compounds (mg/g DW)	White Mugwort Powder (P)												
	P-MetOH	P 50 mg/mL				P 100 mg/mL				P 150 mg/mL			
		Gastric	R (%)	Intestinal	BA (%)	Gastric	R (%)	Intestinal	BA (%)	Gastric	R (%)	Intestinal	BA (%)
Quinic acid	ND	0.2 ± 0 B	-	0.8 ± 0 A	-	0.05 ± 0 B	-	0.25 ± 0 B	-	0.2 ± 0.0 B	-	0.2 ± 0 B	-
5-CQA	0.04 ± 0 ns	0.04 ± 0 ns	88.8	ND	0	0.03 ± 0 ns	87.2	ND	0	0.04 ± 0 ns	96.3	ND	0.0
3-CQA	1.4 ± 0.1 A	0.14 ± 0 C	9.7	ND	0	0.4 ± 0 BC	30.1	ND	0	0.6 ± 0.0 B	39.0	ND	0.0
Rutin	0.6 ± 0.0 A	0.2 ± 0 B	32.0	0.2 ± 0 B	35	0.1 ± 0 B	20.7	0.02 ± 0 B	32	0.1 ± 0.0 B	18.0	0.3 ± 0 B	39
Isovitexin	0.4 ± 0.0 A	0.1 ± 0 B	38.0	ND	0	0.1 ± 0 C	21.1	ND	0	0.1 ± 0.0 C	18.0	ND	0.0
Kaempferol	1.2 ± 0.1 A	ND	0	ND	0	ND	0	ND	0	0.2 ± 0.0 B	18.2	0.02 ± 0 C	1.5
3,5-diCQA	4.6 ± 0.2 A	1.0 ± 0 B	21.5	ND	0	0.5 ± 0 C	10.7	0.1 ± 0 D	1.5	0.4 ± 0 CD	9.6	0.1 ± 0 D	1.4
Morin	0.6 ± 0.1 A	0.13 ± 0 B	21.0	ND	0	0.1 ± 0 BC	18.5	ND	0	0.04 ± 0 CD	7.2	0.1 ± 0 BC	11
Quercetin	0.1 ± 0.0 A	0.03 ± 0 B	26.4	ND	0	0.03 ± 0 B	27.6	0.03 ± 0 B	25	0.03 ± 0 B	25.3	0.04 ± 0 B	31

Values with different letters (A–D) within the same row are significantly different (*p* ≤ 0.05). A *p*-value higher than 0.05 is represented by ns (a nonsignificant difference). ND means not detected. R = Recovery of each compound, and BA = Bioaccessibility of each compound. R and BA of quinic acid were shown as (-) meaning not calculated data.

**Table 2:** The changes of the polyphenol compounds of white mugwort extract during in vitro gastrointestinal digestion.

Compounds (mg/g DW)	White Mugwort Extract (FE)												
	FE-MetOH	FE 5 mg/mL				FE 15 mg/mL				FE 30 mg/mL			
		Gastric	R (%)	Intestinal	BA (%)	Gastric	R (%)	Intestinal	BA (%)	Gastric	R (%)	Intestinal	BA (%)
Quinic acid	ND	ND	-	6.1 ± 0 A	-	0.1 ± 0.0 C	-	1.7 ± 0.6 B	-	ND	-	0.7 ± 0.2 BC	-
5-CQA	0.3 ± 0.0 B	0.2 ± 0.0 B	80.6	4.3 ± 0 A	1671	0.3 ± 0.0 B	125	ND	0	0.4 ± 0.1 B	150	ND	0.00
3-CQA	22.3 ± 2.3 A	11 ± 3.9 CD	50.5	ND	0	17 ± 1.0 B	77.5	8 ± 1. D	36.0	20 ± 2.3 AB	90.6	13.0 ± 0 C	58.4
Rutin	1.8 ± 0.0 B	2.1 ± 0.2 A	122	ND	0	1.0 ± 0.1 D	55.6	1.2 ± 0 CD	68.5	1.3 ± 0.1 C	73.9	1.0 ± 0.1 D	56.2
Isovitexin	0.9 ± 0.0 B	1.5 ± 0.4 A	167	ND	0	0.6 ± 0.2 C	60.8	ND	0	0.7 ± 0.1 BC	80.1	0.4 ± 0.0 C	46.9
Kaempferol	5.3 ± 1.0 B	ND	0	ND	0	11.2 ± 5.7 A	210	1.0 ± 0.2 B	18.8	13.3 ± 5.7 A	249	5.3 ± 0.2 B	99.0
3,5-diCQA	19.9 ± 6.1 A	19 ± 7.0 AB	95.3	1.0 ± 0 C	3.9	14.2 ± 2.6 B	71.6	0.9 ± 0.1 C	4.3	16 ± 4.4 AB	82.2	3.1 ± 0.1 C	15.7
Morin	2.2 ± 0.1 A	1.1 ± 0.2 BC	52.6	ND	0	1.2 ± 0.8 BC	52.9	0.6 ± 0.1 C	25.4	1.7 ± 0.3 AB	76.4	0.6 ± 0.1 C	29.1
Quercetin	0.7 ± 0.0 AB	0.7 ± 0.2 A	106	ND	0	0.3 ± 0 BC	40.5	0.3 ± 0 AB	51.8	0.4 ± 0.1 AB	53.1	0.1 ± 0.0 C	12.7

Values with different letters (A–D) within the same row are significantly different (*p* ≤ 0.05). ND means not detected. R = Recovery of each compound, and BA = Bioaccessibility of each compound. R and BA of quinic acid were shown as (-) meaning not calculated data.

## Data Availability

Data is contained within the article or supplementary material.

## Supplementary Materials

The following supporting information can be downloaded at: https://www.mdpi.com/article/10.3390/foods12050949/s1: Figure S1: Changes in the proportionate total phenolic content release (mg GAE/g DW) from white mugwort powder at different concentrations (P 50, 100, and 150 mg/mL) (A) and white mugwort extract at different concentrations (FE 5, 15, and 30 mg/mL) (B) during in vitro gastrointestinal digestion. Figure S2: Changes in the DPPH scavenging activity (mg GAE/g DW) of white mugwort powder at different concentrations (P 50, 100, and 150 mg/mL) (A) and white mugwort extract at different concentrations (FE 5, 15, and 30 mg/mL) (B) during in vitro gastrointestinal digestion. Figure S3: Changes in the FRAP value (mol Fe(ll)/g DW) of white mugwort powder at different concentrations (P 50, 100, and 150 mg/mL) (A) and white mugwort extract at different concentrations (FE 5, 15, and 30 mg/mL) (B) during in vitro gastrointestinal digestion. Table S1: The contents of polyphenol compounds (mg/g DW) of dried powder and fresh aerial part extract from white mugwort.

## References

[1] Kumar S., Pandey A.K. (2013). Chemistry and Biological Activities of Flavonoids: An Overview. Sci. World J..

[2] Nakamura Y., Ohto Y., Murakami A., Jiwajinda S., Ohigashi H. (1998). Isolation and Identification of Acetylenic Spiroketal Enol Ethers from *Artemisia lactiflora* as Inhibitors of Superoxide Generation Induced by a Tumor Promoter in Differentiated HL-60 Cells. J. Agric. Food Chem..

[3] Ma L., Ge F., Tang C.-P., Ke C.-Q., Li X.-Q., Althammer A., Ye Y. (2011). The absolute configuration determination of naturally occurring diacetylenic spiroacetal enol ethers from *Artemisia lactiflora*. Tetrahedron.

[4] Xiao M.-T., Luo D.-W., Ke Z., Ye J., Tu P.-F. (2014). A novel polyacetylene from the aerial parts of Artemisia lactiflora. Phytochem. Lett..

[5] Lin F.-D., Luo D.-W., Ye J., Xiao M.-T. (2014). Chemical constituents of Artemisia lactiflora(II). China J. Chin. Mater. Med..

[6] Namgoong S., Son K., Chang H., Kang S., Kim H. (1994). Effects of naturally occurring flavonoids on mitogen-induced lymphocyte proliferation and mixed lymphocyte culture. Life Sci..

[7] Kulprachakarn K., Pangjit K., Paradee N., Srichairatanakool S., Rerkasem K., Ounjaijean S. (2019). Antioxidant Properties and Cytotoxicity of White Mugwort (*Artemisia lactiflora*) Leaf Extract in Human Hepatocellular Carcinoma Cell Line. Walailak J. Sci. Technol. (WJST).

[8] Sea-tan S., Kunpanya P. (2017). Water extract from leaf and stem of White Mugwort inhibits enzyme activity of α-amylase and α-glucosidase. J. Nutr. Assoc. Thail..

[9] Udomwasinakun N., Pirak T., Chanput W.P. (2022). Identification of polyphenols in white mugwort (*Artemisia lactiflora* Wall.) ethanolic extracts and their anti-inflammatory and anti-adipogenic activity potential. Food Biosci..

[10] Kim H.K., Cheon B.S., Kim Y.H., Kim S.Y., Kim H.P. (1999). Effects of naturally occurring flavonoids on nitric oxide production in the macrophage cell line RAW 264.7 and their structure–activity relationships. Biochem. Pharmacol..

[11] Gomes A., Fernandes E., Lima J., Mira L., Corvo M. (2008). Molecular Mechanisms of Anti-Inflammatory Activity Mediated by Flavonoids. Curr. Med. Chem..

[12] Schwartz A., Middleton E. (1984). Comparison of the effects of quercetin with those of other flavonoids on the generation and effector function of cytotoxic T lymphocytes. Immunopharmacology.

[13] Khalilpourfarshbafi M., Gholami K., Murugan D.D., Sattar M.Z.A., Abdullah N.A. (2018). Differential effects of dietary flavonoids on adipogenesis. Eur. J. Nutr..

[14] Matsuda H., Kogami Y., Nakamura S., Sugiyama T., Ueno T., Yoshikawa M. (2011). Structural requirements of flavonoids for the adipogenesis of 3T3-L1 cells. Bioorg. Med. Chem..

[15] Liang N., Kitts D.D. (2016). Role of Chlorogenic Acids in Controlling Oxidative and Inflammatory Stress Conditions. Nutrients.

[16] Narita Y., Inouye K. (2013). Degradation Kinetics of Chlorogenic Acid at Various pH Values and Effects of Ascorbic Acid and Epigallocatechin Gallate on Its Stability under Alkaline Conditions. J. Agric. Food Chem..

[17] Naveed M., Hejazi V., Abbas M., Kamboh A.A., Khan G.J., Shumzaid M., Ahmad F., Babazadeh D., FangFang X., Modarresi-Ghazani F. (2018). Chlorogenic acid (CGA): A pharmacological review and call for further research. Biomed. Pharmacother..

[18] Vilas-Boas A.A., Oliveira A., Jesus D., Rodrigues C., Figueira C., Gomes A., Pintado M. (2020). Chlorogenic acids composition and the impact of in vitro gastrointestinal digestion on espresso coffee from single-dose capsule. Food Res. Int..

[19] Minekus M., Alminger M., Alvito P., Ballance S., Bohn T., Bourlieu C., Carrière F., Boutrou R., Corredig M., Dupont D. (2014). A standardised static in vitro digestion method suitable for food—An international consensus. Food Funct..

[20] Czubinski J., Wroblewska K., Czyzniejewski M., Górnaś P., Kachlicki P., Siger A. (2018). Bioaccessibility of defatted lupin seed phenolic compounds in a standardized static in vitro digestion system. Food Res. Int..

[21] Tagliazucchi D., Verzelloni E., Bertolini D., Conte A. (2010). In vitro bio-accessibility and antioxidant activity of grape polyphenols. Food Chem..

[22] Kim Y.E., Ryu J., Kim J.T., Suh S., Hong G.-P., Ko S. (2017). Physicochemical and in vitro digestion characteristics of size-different red ginseng powders. Food Sci. Biotechnol..

[23] Holland C., Ryden P., Edwards C.H., Grundy M.M.-L. (2020). Plant Cell Walls: Impact on Nutrient Bioaccessibility and Digestibility. Foods.

[24] Oh J.-H., Lee C.-Y., Lee Y.-E., Yoo S.-H., Chung J.-O., Rha C.-S., Park M.-Y., Hong Y.-D., Shim S.-M. (2021). Profiling of In Vitro Bioaccessibility and Intestinal Uptake of Flavonoids after Consumption of Commonly Available Green Tea Types. Molecules.

[25] Gullon B., Pintado M.E., Fernández-López J., Pérez-Álvarez J.A., Viuda-Martos M. (2015). In vitro gastrointestinal digestion of pomegranate peel (*Punica granatum*) flour obtained from co-products: Changes in the antioxidant potential and bioactive compounds stability. J. Funct. Foods.

[26] Zeng Q., Xu Z., Dai M., Cao X., Xiong X., He S., Yuan Y., Zhang M., Dong L., Zhang R. (2019). Effects of simulated digestion on the phenolic composition and antioxidant activity of different cultivars of lychee pericarp. BMC Chem..

[27] Daly T., Jiwan M.A., O’Brien N.M., Aherne S.A. (2010). Carotenoid Content of Commonly Consumed Herbs and Assessment of Their Bioaccessibility Using an In Vitro Digestion Model. Plant Foods Hum. Nutr..

[28] Kamiloglu S., Capanoglu E. (2013). In vitro gastrointestinal digestion of polyphenols from different molasses (pekmez) and leather (pestil) varieties. Int. J. Food Sci. Technol..

[29] Stanisavljević N., Soković Bajić S., Jovanović Ž., Matić I., Tolinački M., Popović D., Popović N., Terzić-Vidojević A., Golić N., Beškoski V. (2020). Antioxidant and Antiproliferative Activity of Allium ursinum and Their Associated Microbiota During Simulated in vitro Digestion in the Presence of Food Matrix. Front. Microbiol..

[30] Gião M.S., Gomes S., Madureira A.R., Faria A., Pestana D., Calhau C., Pintado M.E., Azevedo I., Malcata F.X. (2012). Effect of in vitro digestion upon the antioxidant capacity of aqueous extracts of Agrimonia eupatoria, Rubus idaeus, *Salvia* sp. and Satureja montana. Food Chem..

[31] Gonçalves G.A.S., Resende N.S., Carvalho E.E.N., de Resende J.V., Boas E.V.D.B.V. (2017). Effect of pasteurisation and freezing method on bioactive compounds and antioxidant activity of strawberry pulp. Int. J. Food Sci. Nutr..

[32] Gutiérrez-Grijalva E.P., Angulo-Escalante M.A., León-Félix J., Heredia J.B. (2017). Effect of In Vitro Digestion on the Total Antioxidant Capacity and Phenolic Content of 3 Species of Oregano (*Hedeoma patens*, *Lippia graveolens*, *Lippia palmeri*). J. Food Sci..

[33] Zhang A., Wan L., Wu C., Fang Y., Han G., Li H., Zhang Z., Wang H. (2013). Simultaneous Determination of 14 Phenolic Compounds in Grape Canes by HPLC-DAD-UV Using Wavelength Switching Detection. Molecules.

[34] Khochapong W., Ketnawa S., Ogawa Y., Punbusayakul N. (2021). Effect of in vitro digestion on bioactive compounds, antioxidant and antimicrobial activities of coffee (*Coffea arabica* L.) pulp aqueous extract. Food Chem..

[35] Ortega-Vidal J., Ruiz-Riaguas A., Córdova M.F.-D., Ortega-Barrales P., Llorent-Martínez E. (2019). Phenolic profile and antioxidant activity of *Jasonia glutinosa* herbal tea. Influence of simulated gastrointestinal in vitro digestion. Food Chem..

[36] Brodkorb A., Egger L., Alminger M., Alvito P., Assunção R., Ballance S., Bohn T., Bourlieu-Lacanal C., Boutrou R., Carrière F. (2019). INFOGEST static in vitro simulation of gastrointestinal food digestion. Nat. Protoc..

[37] Campoli S.S., Rojas M.L., Amaral J.E.P.G.D., Canniatti-Brazaca S.G., Augusto P.E.D. (2018). Ultrasound processing of guava juice: Effect on structure, physical properties and lycopene in vitro accessibility. Food Chem..

[38] Karabegović I., Nikolova M., Veličković D., Stojičević S., Veljković V., Lazić M. (2011). Comparison of Antioxidant and Antimicrobial Activities of Methanolic Extracts of the *Artemisia* sp. Recovered by Different Extraction Techniques. Chin. J. Chem. Eng..

[39] Hwang K.-E., Choi Y.-S., Choi J.-H., Kim H.-Y., Kim H.-W., Lee M.-A., Chung H.-K., Kim C.-J. (2011). Effect of Ganghwayakssuk (*Artemisia princeps* Pamp.) on oxidative stability of deep fried chicken nuggets. Food Sci. Biotechnol..

[40] Ketnawa S., Suwannachot J., Ogawa Y. (2019). In vitro gastrointestinal digestion of crisphead lettuce: Changes in bioactive compounds and antioxidant potential. Food Chem..

[41] Sivapalan T., Melchini A., Coode-Bate J., Needs P.W., Mithen R.F., Saha S. (2019). An LC-MS/MS Method to Measure S-Methyl-l-Cysteine and S-Methyl-l-Cysteine Sulfoxide in Human Specimens Using Isotope Labelled Internal Standards. Molecules.

[42] Bennouna D., Avice J.-C., Rosique C., Svilar L., Pontet C., Trouverie J., Fine F., Pinochet X., Fraser K., Martin J.-C. (2019). The impact of genetics and environment on the polar fraction metabolome of commercial Brassica napus seeds: A multi-site study. Seed Sci. Res..

[43] von Roepenack-Lahaye E., Degenkolb T., Zerjeski M., Franz M., Roth U., Wessjohann L., Schmidt J., Scheel D., Clemens S. (2004). Profiling of Arabidopsis Secondary Metabolites by Capillary Liquid Chromatography Coupled to Electrospray Ionization Quadrupole Time-of-Flight Mass Spectrometry. Plant Physiol..

[44] Nignpense B.E., Latif S., Francis N., Blanchard C., Santhakumar A.B. (2022). The impact of simulated gastrointestinal digestion on the bioaccessibility and antioxidant activity of purple rice phenolic compounds. Food Biosci..

[45] Yang I., Jayaprakasha G.K., Patil B. (2018). In vitro digestion with bile acids enhances the bioaccessibility of kale polyphenols. Food Funct..

[46] Durdun C., Papuc C., Nicorescu V., Gajaila I., Goran G., Petcu C., Stefan G. (2016). The Influence of Solid-to-Solvent Ratio and Extraction Method on Total Phenolic Content, Flavonoid Content and Antioxidant Properties of Some Ethanolic Plant Extracts. Rev. Chim..

[47] Sęczyk Ł., Sugier D., Świeca M., Gawlik-Dziki U. (2020). The effect of in vitro digestion, food matrix, and hydrothermal treatment on the potential bioaccessibility of selected phenolic compounds. Food Chem..

[48] Zhang Y., Wu S., Qin Y., Liu J., Liu J., Wang Q., Ren F., Zhang H. (2018). Interaction of phenolic acids and their derivatives with human serum albumin: Structure–affinity relationships and effects on antioxidant activity. Food Chem..

[49] Li D.-L., Zheng X.-L., Duan L., Deng S.-W., Ye W., Wang A.-H., Xing F.-W. (2017). Ethnobotanical survey of herbal tea plants from the traditional markets in Chaoshan, China. J. Ethnopharmacol..

[50] Liang Y., Abbott D., Howard N., Lim K., Ward R., Elgendi M. (2019). How Effective Is Pulse Arrival Time for Evaluating Blood Pressure? Challenges and Recommendations from a Study Using the MIMIC Database. J. Clin. Med..

[51] Wahyuni T.S., Purwanto K.K. (2020). Students’ conceptual understanding on acid-base titration and its relationship with drawing skills on a titration curve. J. Phys. Conf. Ser..

[52] Veiga M., Costa E.M., Silva S., Pintado M. (2018). Impact of plant extracts upon human health: A review. Crit. Rev. Food Sci. Nutr..

[53] Bibi Sadeer N., Montesano D., Albrizio S., Zengin G., Mahomoodally M.F. (2020). The Versatility of Antioxidant Assays in Food Science and Safety—Chemistry, Applications, Strengths, and Limitations. Antioxidants.

[54] Francenia Santos Sánchez N., Salas-Coronado R., Villanueva-Cañongo C., Hernández-Carlos B. (2019). Antioxidant Compounds and Their Antioxidant Mechanism. Antioxidants.

[55] Farah A., Donangelo C.M. (2006). Phenolic compounds in coffee. Braz. J. Plant Physiol..

[56] Meinhart A.D., Damin F.M., Caldeirão L., da Silveira T.F.F., Filho J.T., Godoy H.T. (2017). Chlorogenic acid isomer contents in 100 plants commercialized in Brazil. Food Res. Int..

[57] Olennikov D.N., Chirikova N.K., Kashchenko N.I., Nikolaev V.M., Kim S.-W., Vennos C. (2018). Bioactive Phenolics of the Genus *Artemisia* (Asteraceae): HPLC-DAD-ESI-TQ-MS/MS Profile of the Siberian Species and Their Inhibitory Potential Against α-Amylase and α-Glucosidase. Front. Pharmacol..

[58] Chao P.-Y., Lin S.-Y., Lin K.-H., Liu Y.-F., Hsu J.-I., Yang C.-M., Lai J.-Y. (2014). Antioxidant Activity in Extracts of 27 Indigenous Taiwanese Vegetables. Nutrients.

[59] Booth A.N., Emerson O., Jones F.T., DeEds F. (1957). Urinary metabolites of caffeic and chlorogenic acids. J. Biol. Chem..

[60] Booth A.N., Williams R.T. (1963). Dehydroxylation of Caffeic Acid by Rat and Rabbit Cæcal Contents and Sheep Rumen Liquor. Nature.

