# Supplementary material for: Assessment of Polyphenols Bioaccessibility, Stability, and Antioxidant Activity of White Mugwort (Artemisia lactiflora Wall.) during Static In Vitro Gastrointestinal Digestion
Source: Foods. 2023 Feb 23;12(5):949. doi: 10.3390/foods12050949 (PMC10000887; doi:10.3390/foods12050949)
Supplement: Supplementary file 1 [file foods-12-00949-s001.zip › foods-2183142-supplementary.pdf]

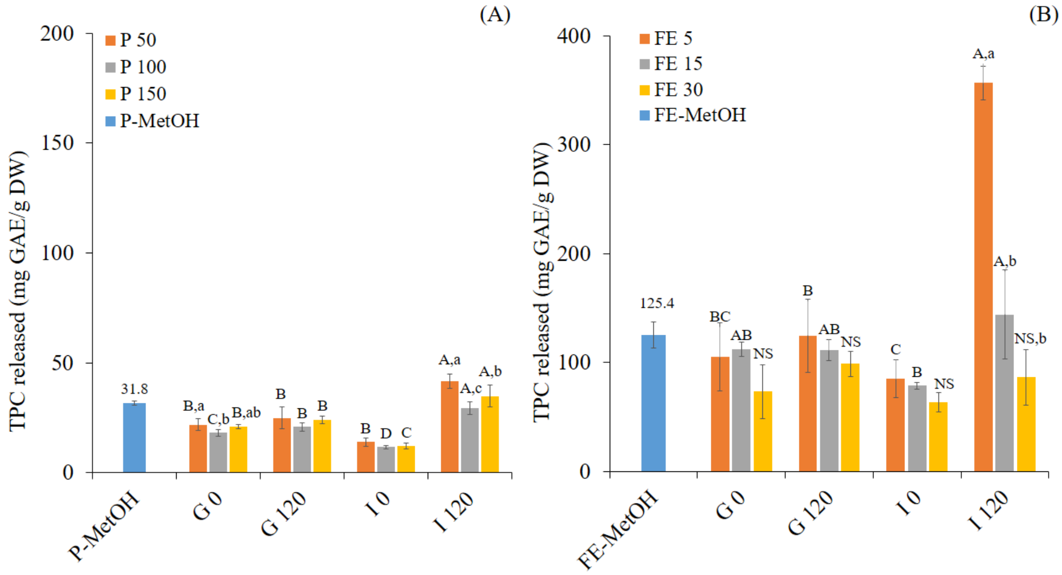

Figure S1. Changes in proportionate total phenolic content release (mg GAE/g DW) from white mugwort powder at different concentrations (P 50, 100, 150 mg/mL) (A) and white mugwort extract at different concentrations (FE 5, 15, 30 mg/mL) (B) during in vitro gastrointestinal digestion. Values with different letters (A-D) within the same treatment are significantly different ($p \leq 0.05$). Values with different letters (a-c) within the same digestion phase are significantly different ($p \leq 0.05$). P-value higher than 0.05 is represented by NS (non-significant difference).

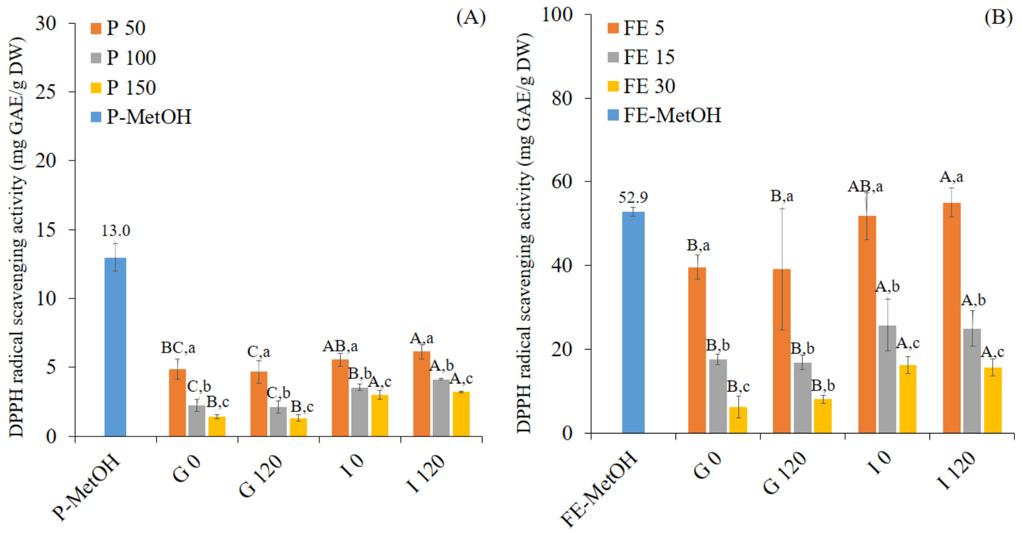

Figure S2. Changes in DPPH scavenging activity (mg GAE/g DW) of white mugwort powder at different concentrations (P 50, 100, 150 mg/mL) (A) and white mugwort extract at different concentrations (FE 5, 15, 30 mg/mL) (B) during in vitro gastrointestinal digestion. Values with different letters (A-C) within the same treatment are significantly different ($p \le 0.05$). Values with different letters (a-c) within the same digestion phase are significantly different ($p \le 0.05$).

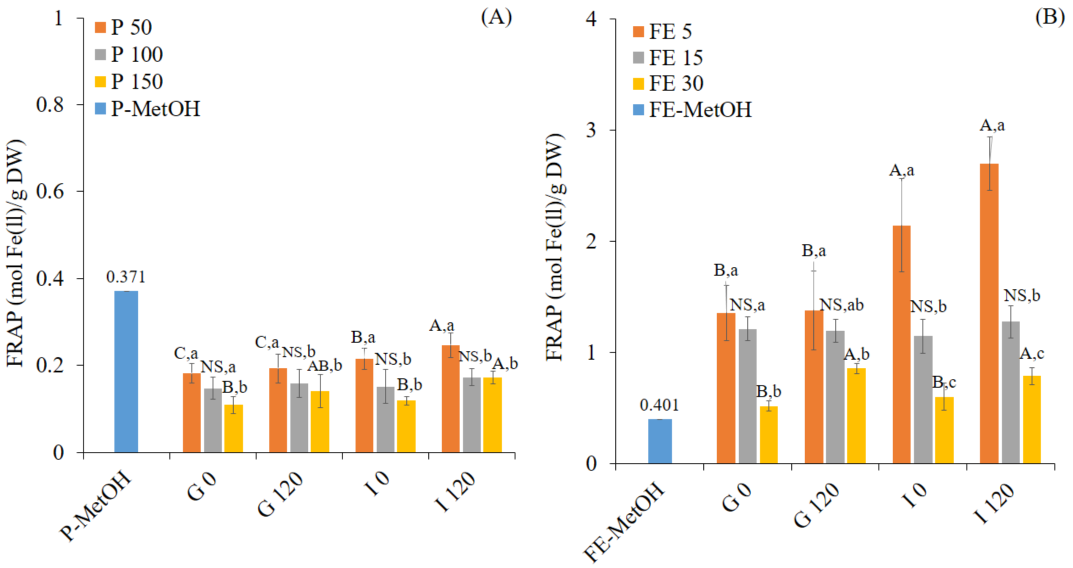

Figure S3 Changes in FRAP value (mol Fe(ll)/g DW) of white mugwort powder at different concentrations (P 50, 100, 150 mg/mL) (A) and white mugwort extract at different concentrations (FE 5, 15, 30 mg/mL) (B) during in vitro gastrointestinal digestion. Values with different letters (A-C) within the same treatment are significantly different ($p \leq 0.05$). Values with different letters (a-c) within the same digestion phase are significantly different ($p \leq 0.05$).

Table S1 The content of polyphenol compounds (mg /g DW) of dried powder and fresh aerial part extract from white mugwort.

No	Compounds (mg/g dry DW)	Retention time (min)	Dried powder (P)	Fresh aerial part extract (FE)
1	5-CQA	12.4	0.04 ± 0.00	0.26 ± 0.01
2	3-CQA	26.7	1.40 ± 0.09	22.26 ± 2.28
3	Sinapoylmalate*	38.2	1265.32 ± 813.53*	8665.15 ± 50.33*
4	Rutin	42.5	0.65 ± 0.02	1.75 ± 0.02
5	Isovitexin	42.9	0.36 ± 0.01	0.92 ± 0.01
6	Kaempferol	47.7	1.21 ± 0.09	5.34 ± 1.03
7	3,5-diCQA	48.8	4.58 ± 0.15	19.85 ± 6.10
8	Morin	61.3	0.60 ± 0.05	2.17 ± 0.12
9	Quercetin	66.8	0.12 ± 0.01	0.66 ± 0.01

*Compounds were identified by mass spectrum compared with literature and the data were expressed as relative peak area.
